# Phytohormone profiles are strongly altered during induction and symptom development of the physiological ripening disorder berry shrivel in grapevine

**DOI:** 10.1007/s11103-020-00980-6

**Published:** 2020-02-18

**Authors:** Michaela Griesser, Stefania Savoi, Suriyan Supapvanich, Petre Dobrev, Radomira Vankova, Astrid Forneck

**Affiliations:** 1 Department of Crop Sciences, Institute of Viticulture and Pomology, University of Natural Resources and Life Sciences, Konrad Lorenz Straße 24, Tulln, 3430 Vienna, Austria; 2 AGAP, Montpellier University, CIRAD, INRA, Montpellier SupAgro, 2 Place Pierre Viala, 34060 Montpellier, France; 3 Department of Agricultural Education, Faculty of Industrial Education and Technology, King Mongkut’s Institute of Technology Ladkrabang, 1 Chalongkrung Road, Ladkrabang, Bangkok, 10520 Thailand; 4 Institute of Experimental Botany, Czech Academy of Sciences, Prague, Czech Republic

**Keywords:** Grapevine, Ripening regulation, Physiological disorders, Phytohormones

## Abstract

The process of grape berry ripening follows three phases with distinct metabolic processes and complex regulations via phytohormones. The physiological ripening disorder berry shrivel (BS) is characterized by reduced sugar accumulation, low anthocyanin contents, and high acidity in affected berries. The processes leading to BS induction are unknown, but recent transcriptional data on reduced expression of switch genes hint towards a disturbed ripening onset. Herein we investigated the phytohormone composition throughout grape berry ripening in healthy and BS berries in *Vitis vinifera* L. cultivar Blauer Zweigelt. Thereby we hypothesize that phytohormones are key players for BS induction and suppress the expression of switch genes at veraison. The presented metabolomics and RNAseq data describe two distinct phytohormone profiles in BS berries, differing between pre- and post-veraison with a clear ethylene precursor (aminocyclopropane-1-carboxylic acid, ACC) peak before veraison. Exogenous application of ACC led to BS symptoms, while ethephone application led to berry abscission. During post-veraison, we observed high ABA-glucose ester (ABA-GE) and low indole-3-acetate aspartate (IAA-Asp) and isopentenyladenine (iP) contents in BS berries and the transcriptional induction of several phytohormone pathways. The presented descriptive data provide valuable knowledge to further decipher the role of phytohormones in BS induction and BS symptom development.

**Electronic supplementary material:**

The online version of this article (10.1007/s11103-020-00980-6) contains supplementary material, which is available to authorized users.

## Key message

Ripening disorder berry shrivel in grapevine leads to distinct phytohormone pattern pre- and post-veraison in grape berries.

**Electronic supplementary material:**

The online version of this article (10.1007/s11103-020-00980-6) contains supplementary material, which is available to authorized users.

## Introduction

Sugar accumulation disorder, known as berry shrivel (BS), is a grapevine ripening disorder with still unclear physiological mechanisms. Grape berry quality and yield are highly affected by this disorder and BS grape clusters are not considered for winemaking. All shriveling disorders in grapevine like sunburn, late-season dehydration, bunch stem necrosis, and BS have the common feature of shrinking berries, but the time of emergence, the morpho-anatomical characteristics and the metabolite content are distinct from each other (Bondada and Keller 2012a; Griesser et al. 2012; Krasnow et al. 2010). Recognizable symptoms of BS are: a stop of sugar accumulation short after veraison, high contents of organic acids, low pH values and low amounts of anthocyanins in red grape varieties (Griesser et al. 2018; Savoi et al. 2019). Dissimilar from bunch stem necrosis, rachis and pedicels stay viable and green with no obvious symptoms on the surface. Nevertheless morpho-anatomical study observed cell death in the rachis of symptomatic grape clusters of Cabernet Sauvignon (Bondada and Keller 2012b; Hall et al. 2011), TEM analysis showed collapsed cells and cell wall thickenings in the secondary phloem (Crespo-Martinez et al. 2019) and light microscopy and SEM analyses revealed higher rates of callose deposition on sieve plates (Bondada 2016; Crespo-Martinez et al. 2019). Reduced cell viability was also observed in grape berries leading to the assumption that the loss of cell membrane integrity is an important factor for water loss and berry shrinkage (Krasnow et al. 2008, 2012). The major drawback of these studies is the use of already symptomatic grape clusters, which prevents distinction of causal BS inducing events from the follow up symptoms. Recently, it was shown that transcriptional changes occur in BS grape berries at the onset of ripening before symptoms are visible, while no alterations in berry transcriptome were observed in pre-veraison samples (Griesser et al. 2017; Savoi et al. 2019). Specifically, a subset of genes, called switch genes, described as master regulators of the ripening onset in grape berries (Massonnet et al. 2017; Palumbo et al. 2014), were down-regulated in BS berries at veraison, suggesting an altered ripening induction (Savoi et al. 2019). Later ripening stages showed more than 3000 genes differentially expressed, among them up-regulation of genes related to phytohormone biosynthesis, response to stress and phenylpropanoid pathway, whereas genes related to the flavonoid pathway and the sugar metabolism were expressed to a lower extend in BS grapes (Savoi et al. 2019). In conclusion, low anthocyanin and sugar content are likely the consequences of a disturbance of the entire ripening process as indicated by the lower expression of several switch genes at veraison in BS berries. Phytohormone metabolites are possible candidates for switch gene regulation and many of the switch genes are assumed to be regulated by abscisic acid (ABA) (Pilati et al. 2017; Savoi et al. 2019).

The process of grape berry development and ripening displays a double sigmoid growth curve with three distinct phases (Coombe and Hale 1973). Veraison marks the initiation of sugar accumulation and the rapid pigmentation of berries by anthocyanins in red grape varieties. Grape berry ripening, especially the transition from the first growth phase to ripening related processes later in development, is very complex and just recently this complexity was revealed by applying omics techniques (Fasoli et al. 2018; Pilati et al. 2017). In general, it is assumed that ABA, ethylene, and brassinosteroids provide the signals for the onset of ripening although the processes are unclear as previously reviewed (Fortes et al. 2015; Kuhn et al. 2014b). Especially the role of ethylene in combination with other phytohormones is under debate (Chervin et al. 2006; Sun et al. 2010). Additionally, physical modifications prior to the ABA signal start the softening process of berries via loss of cell turgor and elasticity (Castellarin et al. 2016). Yet not fully understood, it is possible that an interplay takes place between ethylene and ABA during the ripening phase (Sun et al. 2010). Auxin is a negative regulator of berry ripening, as high contents are observed in green fast growing berries whereas very low levels are detected at veraison and throughout the ripening period (Bottcher et al. 2010). Exogenous artificial auxin application delayed ripening (Bottcher et al. 2011), along with a lower expression of genes related to the ABA biosynthesis and signaling pathways, while ethylene biosynthesis was induced (Ziliotto et al. 2012).

Apart from metabolic signals, a timeline of events leading to the onset of ripening was suggested with an initial fall of berry elasticity and turgor pressure followed by ABA and sugar accumulation and coloring pigments in skins of red grape varieties (Castellarin et al. 2016). More specifically two rapid transcriptional transitions starting 14 d before veraison have been characterized in grape berries, suggesting a hierarchy of signals for the onset of ripening (Fasoli et al. 2018). BS berries have low sugar contents and reduced anthocyanin biosynthesis (Griesser et al. 2018), suggesting a disturbed ripening process, which is supported by the transcriptional repression of switch genes in BS berries at veraison (Savoi et al. 2019). The phytohormone crosstalk regulating grape berry ripening is not fully understood, but there is a strong evidence that ABA, brassinosteroids and ethylene induce and promote ripening, while auxin can delay the major ripening associated processes. Here we firstly describe the phytohormone composition and linked gene expression of healthy and BS-grapes during the ripening period of two vintages on *V. vinifera* cv. Zweigelt. We hypothesize that ethylene and its crosstalk with other hormones induces BS in grape berries. In general, ripening control by phytohormones needs further attention, like timing, sensitivity of the tissue, molecules involved and their concentrations.

## Materials and methods

### Plant material and sampling

**Fig. 1:**
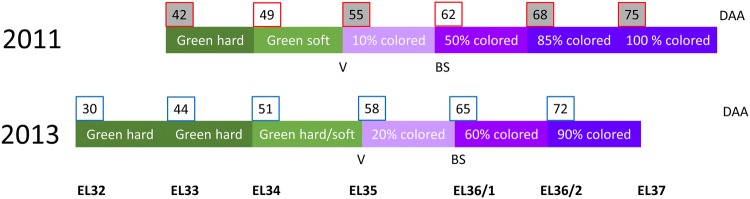
Sampling scheme for 2011 and 2013 with observed phenology, EL categorization and days after anthesis (DAA). Grape berry development and ripening were slightly different between years but substantial overlaps were obtained for comparison. Samples for phytohormone and qPCR analyses (grey background) were obtained at EL33, EL35, EL36/2 and EL37 corresponding to 42, 55, 68 and 75 DAA collected 2011. RNA Seq analyses and qPCR were performed using samples from 2013. Veraison (V) is stated as the onset of coloring and the first BS symptoms were observed at EL36/1 in both years

Berry samples of the red grape cultivar Zweigelt (*V. vinifera*), grafted on rootstock Kober 5BB, were collected in 2011 and 2013 from a commercial vineyard located in Lower Austria (Antlasberg, Mailberg GPS coordinates 48.6667, 16.1833; yearly climate conditions are shown in Figure S1) as previously described (Griesser et al. 2018, 2017; Savoi et al. 2019). In both years, grape bunches were randomly labeled after anthesis and samples were obtained in 2011 from EL33 (BBCH79) till EL38 (BBCH89) and in 2013 from EL32 (BBCH77) till EL37 (BBCH89). This corresponds to 30–75 days after anthesis (DAA) as shown in Fig. 1. Samples were obtained from each labeled cluster once to allow an adequate a posteriori categorization into healthy (H, control) or BS-affected (BS) ones by not influencing the ripening process. Sugar accumulation and anthocyanin biosynthesis have been described for H and BS samples elsewhere (Griesser et al. 2018; Savoi et al. 2019). Three biological replicates for each sampling point and condition were considered for all analyses conducted. Each biological replicate resulted from the pooling of three different bottom clusters and only samples which were clearly categorized into the categories “healthy, control” and “BS” were used for further analyses. BS is affecting whole clusters in case of Zweigelt, therefore the obtained samples are representative for the cluster. Before analyses, frozen berries were ground to a fine powder under liquid nitrogen using a ball mill (Retsch MM400).

### Phytohormone quantification

Four sampling points (42, 55, 68, 75 DAA corresponding to EL33, EL35, EL36/2, and EL37) from 2011 were selected to determine phytohormones in whole berries according to a method previously described (Dobrev and Kaminek 2002; Dobrev and Vankova 2012). In short, approximately 50 mg (FW) samples were homogenized and extracted with cold (− 20 °C) methanol/water/formic acid (15/4/1, v/v/v). To account for sample losses and for quantification by isotope dilution, the following isotope-labelled internal standards (10 pmol/sample) were added: ^13^C_6_-IAA (Cambridge Isotope Laboratories, Tewksbury, MA, USA), ^2^H_4_-SA (Sigma Aldrich, St. Louis, MO, USA), ^2^H_5_-JA (Olchemim, Olomouc, Czech Republic), ^2^H_3_-PA, ^2^H_3_-DPA (NRC-PBI, Saskatoon, Canada), ^2^H_6_-ABA, ^2^H_5_-JA, ^2^H_2_-GA_4_, ^2^H_5_-*trans*Z, ^2^H_5_-*trans*ZR, ^2^H5-*trans*Z7G, ^2^H_5_-*trans*Z9G, ^2^H_5_-*trans*ZOG, ^2^H_5_-*trans*ZROG, ^2^H_5_-*trans*ZRMP, ^2^H_3_-DZ, ^2^H_3_-DZR, ^2^H_3_-DZ9G, ^2^H_6_-iP, ^2^H_6_-iPR, ^2^H_6_-iP7G, ^2^H_6_-iP9G, ^2^H_6_-iPRMP (Olchemim, Olomouc, Czech Republic). The extract was applied to a mixed mode reverse phase–cation exchange SPE column (Oasis-MCX, Waters, Milford, MA, USA). Hormone fraction eluted with methanol contained hormones of acidic character [auxins, ABA, salicylic acid (SA), jasmonic acid (JA), gibberellins (GA)]. The basic fraction was eluted with 0.35 M NH_4_OH in 60% methanol (cytokinins, ACC). Fractions were evaporated to dryness in a vacuum concentrator and dissolved into 30 µL of 10% methanol. An aliquot (10 µL) was analyzed using a high-performance liquid chromatography (HPLC) (Ultimate, 3000 Dionex, Sunnyvale, USA) coupled to a hybrid triple quadrupole/linear ion trap mass spectrometer (3200 Q TRAP, Applied Biosystems, Foster City, USA) set in selected reaction monitoring mode. Chromatographic conditions included a HPLC column Luna C18(2) (100 × 2 mm, 3 µm, Phenomenex, Torrance, USA) at flow rate of 0.25 mL min^−1^. Quantification of hormones was done using the isotope dilution method with multilevel calibration curves (r^2^ > 0.99). Data processing was carried out with Analyst 1.5 software (Applied Biosystems, Foster City, USA). Final compound determination values were given as absolute concentrations in pmol g^−1^ DW.

### RNA extraction and expression analyses

Total RNA from samples 2011 for qPCR analyses was extracted following the protocol previously described (Griesser et al. 2017; Reid et al. 2006), while RNA extraction from samples 2013 was performed with the ‘Spectrum Plant total RNA’ kit (Sigma-Aldrich) for RNASeq analyses.

The following chemicals were used for qPCR analyses: Amplification Grade DNase 1 (Sigma Aldrich) for DNA digestion, GoScript Reverse Transcription System (Promega, Madison, WI, USA) for cDNA synthesis, 2X KAPA SYBR FAST qPCR Universal (Peqlab, Erlangen, Germany) for qPCRs. Cycling conditions were as follows: activation 4 min at 95 °C, 40 cycles for 8 s at 95 °C, 20 s at 60 °C, 30 s at 72 °C and 5 s at 75 °C with fluorescence measurement. The reference genes actin (VIT_04s0044g00580) and ef1 (VIT_06s0004g03220) were used for qPCR and data were calculated as NRQ (Hellemans et al. 2007) with the R-package “EasyqpcR”. List of primers used is shown in Table S1.

### Induction of BS by exogenous ethylene application

The phytohormone quantification showed a strong increase of ACC in BS berries before veraison. The effect of exogenous ethylene application on BS incidence was tested by applying four different treatments: control (water spraying), 1500 mg L^−1^ Ethephon (2-chloroethylphosphonic acid, Bayer Crop Science), 1000 mg L^−1^ ACC (1-aminocyclopropane-1-carboxylic acid, VBC-30426 Testproduct (10%), Valent BioScience), and 250 mg L^−1^ AVG (l-α-(2-aminoethoxyvinyl)glycine hydrochloride, ReTain (15%), Valent BioScience). Chemicals were applied to Zweigelt grape clusters (clone St.9 grafted on Kober 5BB, planted 2011 UFT experimental vineyard, Tulln, Lower Austria). The spraying was performed once with 10 mL solution each (30 grapes per treatment) on the 22.07.2016, 10 days before veraison. All grape clusters were evaluated on the 13.09.2016.

### Statistical analysis

All statistical comparisons were conducted using IBM SPSS Statistics 21. Normal distribution of data sets was tested using the Shapiro–Wilk test. Significant differences were tested by comparison of healthy and BS affected berries with Student’s *t* test (*P* < 0.05) if normal distribution was ensured, otherwise non-parametric Mann–Whitney-U tests were conducted. Heatmaps were drawn using R.

## Results and discussion

The phytohormone profile (metabolite levels and gene expression) of berries from the red grape cultivar Zweigelt in healthy grape clusters (H) and grape clusters developing BS symptoms, a physiological ripening disorder, was analyzed from pre-veraison (pre-symptomatic grape clusters, EL32, EL33, EL34), veraison (EL35) until ripening (with and without BS symptoms, EL36/2, EL37). In total, 45 phytohormone metabolites were detected and quantified at four time points (Table 1). Additionally the transcriptional regulations of phytohormone related genes in H and BS berries obtained from RNASeq analyses (Savoi et al. 2019) are shown as heatmaps and have been validated with qPCR.

**Table 1:** Results of the quantification of phytohormone metabolites with HPLC–MS in healthy (H) and berry shrivel affected (BS) grape clusters collected 42, 55, 68, and 75 days after anthesis (DAA) in 2011

Metabolite (abbreviations)	Days after anthesis (mean value ± standard error; data in pmol g^−1^ DW)							
	42 (EL33)		55 (EL35)		68 (EL36/2)		75 (EL37)	
	Healthy	Berry shrivel	Healthy	Berry shrivel	Healthy	Berry shrivel	Healthy	Berry shrivel
Auxins (AUX)								
Indole-3-actic acid (IAA)	44 ± 10	49 ± 15	39 ± 13	41 ± 8	32 ± 15	36 ± 7	25 ± 2	31 ± 8
IAA-aspartate (IAA-Asp)	135 ± 1 a	89 ± 17 b	134 ± 11	137 ± 34	874 ± 238 a	232 ± 27 b	302 ± 340	228 ± 42
IAA-glutamate (IAA-Glu)	21 ± 10	9 ± 4	12 ± 6	11 ± 6	40 ± 28	19 ± 6	19 ± 15	19 ± 7
oxo-IAA (OxIAA)	713 ± 147 a	144 ± 40 b	176 ± 54	138 ± 15	159 ± 38	132 ± 8	99 ± 83	176 ± 53
Phenylacetic acid (PAA)	2184 ± 268	1554 ± 520	2600 ± 269	2310 ± 368	1296 ± 316	2005 ± 831	1079 ± 444	1202 ± 154
IAA-glucose ester (IAA-GE)	6 ± 3	3 ± 3	2 ± 2	2 ± 1	1 ± 1	0 ± 0	1 ± 1	2 ± 0
Indole-3-pyruvic acid (IPyA)	6 ± 4	5 ± 2	8 ± 7	4 ± 4	6 ± 1	7 ± 4	4 ± 3	10 ± 2
Indole-3-acetamide (IAM)	4 ± 3	5 ± 3	4 ± 3	4 ± 4	4 ± 4	7 ± 4	1 ± 1	6 ± 5
Indole-3-acetonitrile (IAN)	5 ± 3	2 ± 2	4 ± 3	0 ± 0	0 ± 0	0 ± 0	0 ± 0	0 ± 0
Cytokinins (CK)								
Active cytokinins								
*Trans*-zeatin (tZ)	18.8 ± 5.2	8.6 ± 4.5	4.6 ± 1.0	3.9 ± 1.5	19.4 ± 5.8	16.3 ± 4.4	21.9 ± 3.9 a	11.9 ± 2.3 b
*Cis*-zeatin (cZ)	1.6 ± 2.1	1.1 ± 0.9	1.3 ± 0.6	1.1 ± 0.6	0.7 ± 0.6	1.1 ± 0.6	6.9 ± 2.8 a	0.4 ± 0.2 b
Dihydrozeatin (dZ)	19.8 ± 6.8	8.4 ± 2.0	6.7 ± 3.6	3.4 ± 1.0	24.4 ± 9.0 a	3–4 ± 0.6 b	1.5 ± 0.9	4.4 ± 1.6
Isopentenyl adenin (iP)	4.9 ± 1.9	1.7 ± 1.0	4.6 ± 2.0	2.8 ± 0.4	9.1 ± 1.1 b	13.8 ± 1.5 a	108.0 ± 5.9 a	5.8 ± 1.1 b
Cytokinin ribosides								
*Trans*-zeatin riboside (tZR)	22.2 ± 6.9	7.7 ± 1.2	6.3 ± 3.6	11.8 ± 1.1	12.2 ± 4.3	2.6 ± 0.7	1.9 ± 1.5	4.3 ± 3.3
*Cis*-zeatin riboside (cZR)	1.3 ± 0.4	1.0 ± 0.2	1.0 ± 0.5	1.1 ± 0.2	0.8 ± 1.0	0.8 ± 0.2	0.3 ± 0.1 b	1.3 ± 0.3 a
Dihydrozeatin riboside (dZR)	15.6 ± 6.1	8.7 ± 2.7	10.9 ± 3.2	8.0 ± 1.0	20.6 ± 5.8 a	2.1 ± 0.8 b	1.4 ± 1.2	1.4 ± 1.2
Isopentenyladenosine (iPR)	1.4 ± 1.3	1.9 ± 0.1	0.7 ± 0.5	0.4 ± 0.6	0.1 ± 0.3	0.4 ± 0.4	0.1 ± 0.2	0 ± 0
Deactivated cytokinins								
*Trans*-zeatin-7-glucoside (tZ7G)	11.2 ± 1.6 a	6.0 ± 0.9 b	3.6 ± 1.2	3.8 ± 1.7	3.5 ± 1.8	2.2 ± 0.7	1.8 ± 0.6	3.7 ± 2.1
*Trans*-zeatin-9-glucoside (tZ9G)	2.0 ± 1.5	0.9 ± 0.5	1.1 ± 0.2	0.6 ± 0.3	0.8 ± 0.6	0.4 ± 0,.3	0.3 ± 0.4	0.2 ± 0.2
Dihydrozeatin-9-glucoside (dZ9G)	11.3 ± 3.3 a	1.4 ± 0.5 b	3.7 ± 1.5	4.1 ± 1.8	7.6 ± 5.6	2.0 ± 0.3	1.8 ± 1.0	3.7 ± 2.8
Isopentenyladenine-7-glucoside (iP7G)	6.3 ± 3.5	1.2 ± 0.4	2.9 ± 1.4	3.4 ± 1.7	1.6 ± 0.7	0.8 ± 0.5	0.6 ± 0.4 b	2.4 ± 0.7 a
Isopentenyladenine-9-glucoside (iP9G)	0.1 ± 0.1	0.1 ± 0.0	0.1 ± 0.1	0.1 ± 0.0	0.2 ± 0.2	0.1 ± 0.1	0.0 ± 0.0	0.2 ± 0.3
Storage cytokinins								
*Trans*-zeatin-*O*-glucoside (tZOG)	40.4 ± 6.6 a	15.7 ± 4.4 b	16.3 ± 7.6	21.9 ± 5.5	19.3 ± 9.2	8.7 ± 2.4	11.2 ± 3.7 b	26.0 ± 2.6 a
*Trans*-zeatin riboside-*O*-glucoside (tZROG)	9.0 ± 5.9	2.5 ± 1.8	3.1 ± 1.7	3.7 ± 3.0	5.7 ± 1.5	5.1 ± 1.0	1.6 ± 1.8	4.5 ± 2.3
Dihydrozeatin riboside -*O*-glucoside (DRZOG)	18.9 ± 7.9 a	3.3 ± 2.1 b	11.2 ± 5.7	11.1 ± 3.8	43.3 ± 13.6 a	1.9 ± 2.1 b	1.3 ± 1.8	1.6 ± 1.8
*Cis*-zeatin-*O*-glucoside (cZOG)	1.2 ± 0.9	0.5 ± 0.3	0.3 ± 0.1	0.3 ± 0.2	0.4 ± 0.2	0.3 ± 0.3	0.3 ± 0.1	0.4 ± 0.4
*Cis*-zeatin riboside -*O*-glucoside (cZROG)	2.2 ± 2.9	1.5 ± 1.6	1.6 ± 1.3	2.0 ± 0.3	1.7 ± 1.2	2.4 ± 3.5	1.0 ± 0.8	1.5 ± 1.6
Cyotkinin precursors								
*Tran*s-zeatin riboside monophosphate (tZRMP)	13.1 ± 3.2 a	2.8 ± 1.1 b	4.2 ± 4.2	2.2 ± 1.5	2.8 ± 1.2	1.1 ± 0.8	1.9 ± 1.4 b	11.1 ± 3.5 a
*Cis*-zeatin riboside monophosphate (cZRMP)	1.5 ± 1.6	0.1 ± 0.1	0.2 ± 0.2	0.5 ± 0.4	0.8 ± 0.5	0.4 ± 0.3	0.3 ± 0.2	0.4 ± 0.4
Dihydrozeatin riboside monophosphate (DZRMP)	13.7 ± 3.2	18.5 ± 0.6	7.0 ± 2.4	4.1 ± 2.5	12.9 ± 7.3 a	0.5 ± 0.5 b	6.8 ± 1.7	2.6 ± 2.8
Isopentenyladenosine monophosphate (iPRMP)	10.4 ± 5.0	5.1 ± 3.5	2.3 ± 1.2	2.1 ± 0.8	2.1 ± 0.9	4.1 ± 3.8	4.2 ± 1.0 a	0.2 ± 0.1 b
Salicylic acid (SA)								
Salicylic acid (SA)	855 ± 114	950 ± 144	625 ± 26 b	683 ± 13 a	423 ± 124	658 ± 161	258 ± 9 b	470 ± 28 a
Jasmonic acid (JA)								
Jasmonic acid (JA)	169 ± 62	154 ± 28	74 ± 16	106 ± 15	32 ± 12	31 ± 1	16 ± 4	31 ± 10
JA-isoleucine (JA-Ile)	15 ± 5	23 ± 9	28 ± 8	27 ± 9	7 ± 3	10 ± 3	4 ± 2	9 ± 6
cisOPDA	144 ± 64	63 ± 10	634 ± 11 a	163 ± 24 b	25 ± 11	22 ± 4	15 ± 18	17 ± 9
Ethylene (ET)								
1-Aminocyclopropane-1-carboxylic acid (ACC)	9387 ± 969 b	29,535 ± 3864 a	5499 ± 1226 b	10,108 ± 1432 a	7610 ± 3045	7455 ± 2468	6037 ± 802 b	12,180 ± 1693 a
Absissic acid (ABA)								
Abscisic acid (ABA)	5232 ± 1615	3411 ± 368	23,698 ± 5039	16,670 ± 4030	15,125 ± 3115	20,515 ± 2430	7085 ± 1247	10,215 ± 1846
ABA-glucose ester (ABA-GE)	4552 ± 265	4828 ± 252	12,528 ± 474 a	6732 ± 622 b	11,941 ± 1672 b	19,460 ± 3475 a	9332 ± 1122 b	23,818 ± 7126 a
Phaseic acid (PA)	30 ± 8	44 ± 10	26 ± 4	26 ± 2	9 ± 2	10 ± 12	10 ± 2	4 ± 4
Dihydrophaseic acid (DPA)	4730 ± 1790	7166 ± 1385	1106 ± 268 a	694 ± 83 b	160 ± 23	202 ± 77	147 ± 36 a	83 ± 11 b
Giberellins								
Giberellin 4 (GA_4_)	25 ± 13	11 ± 5	11 ± 5	8 ± 3	26 ± 11	15 ± 23	10 ± 5	6 ± 3
Giberellin 19 (GA_19_)	51 ± 10 a	26 ± 3 b	27 ± 6	29 ± 3	12 ± 4	23 ± 17	21 ± 2	15 ± 1

Data presented are mean values on dry weight basis ± standard error (*n* = 3 each). Statistical significant differences are indicated with different letters (*P* < 0.05)

### Major induction of ABA pathways during the ripening phase and minor reduction of ABA at veraison in BS berries

**Fig. 2:**
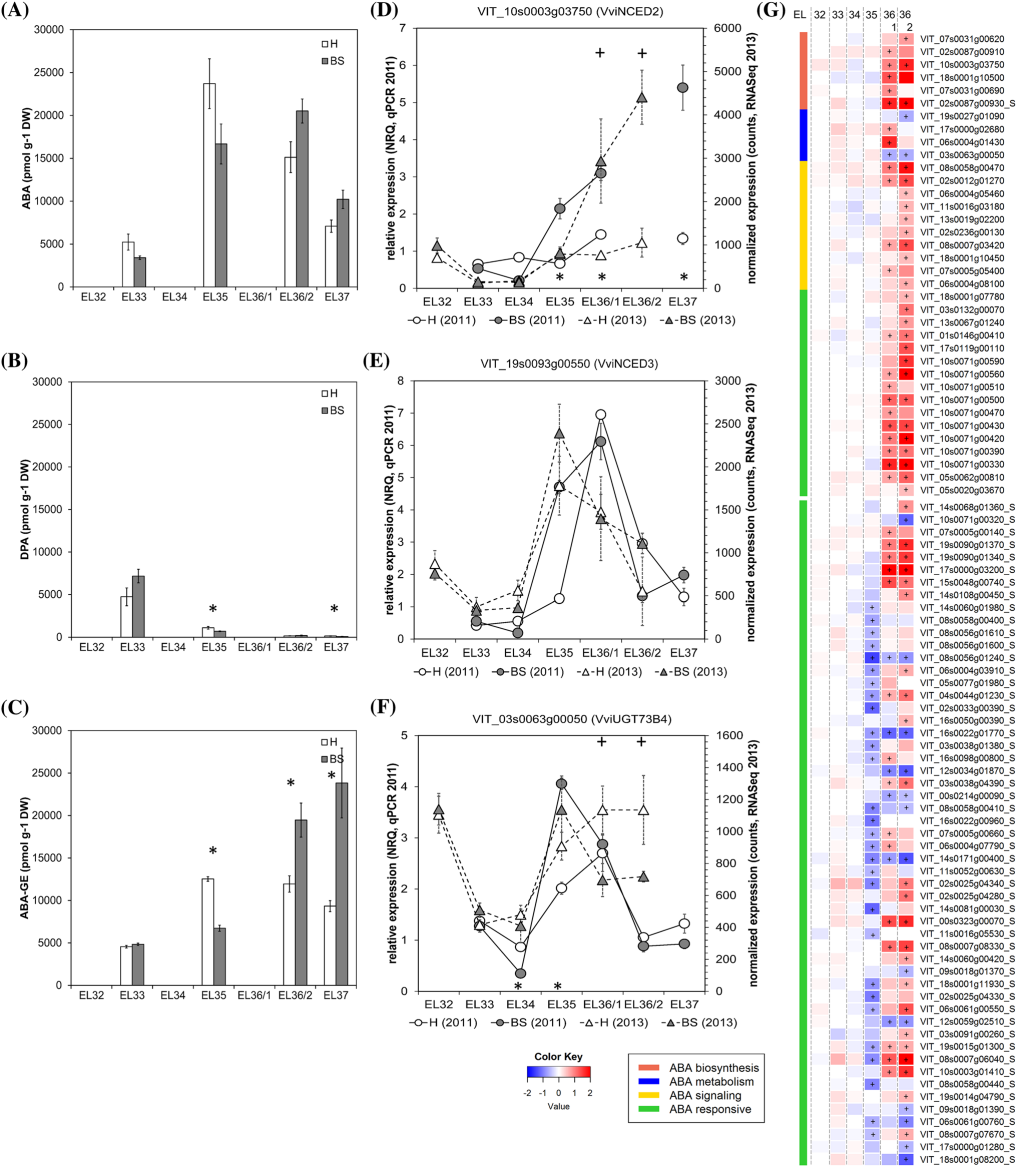
Results obtained from analyses of ABA and its metabolites (**a**–**c**) and expression of ABA metabolism- and signal transduction-related genes in healthy (H) and berry shrivel (BS) grape clusters collected at six sampling dates (EL32, EL33, EL34, EL35, EL36/1, EL36/2, EL37) (**d**–**g**). **a** Abscisic acid (ABA) content, **b** phaseic acid content (DPA) and **c** ABA-glucose ester content (ABA-GE) in berry samples collected 2011. Expression of ABA biosynthetic genes genes *VviNCED2* (**d**) and *VviNCED3* (**e**) and ABA-metabolic gene *VviUGT73B4* (**f**) determined by qPCR (samples 2011) in comparison with RNAseq analyses (samples 2013). **g** RNAseq results on genes related to ABA biosynthesis, metabolism, signaling, and responses in BS samples shown as logFC. All data are mean values ± standard error (*n* = 3 each).Veraison (V) is defined as the start of coloring (EL35) and first BS symptoms were determined at EL36/1. Statistical significant differences are indicated with an asterisk (2011) and a plus symbol (2013) (*P* < 0.05)

Abscisic acid (ABA) and its three metabolites ABA glucose ester (ABA-GE), phaseic acid (PA), and dihydrophaseic acid (DPA) were quantified in H and BS berries (Table 1). Our data (Fig. 2a) show an increase of ABA at veraison (EL35) in both H and BS berries and its decrease during the ripening phase (EL37). These results are in accordance with previous studies (Castellarin et al. 2016; Gambetta et al. 2010; Wheeler et al. 2009). An activation of ABA biosynthesis genes before veraison that could explain the ABA peak at veraison is not observed (Fig. 2g). In accordance, high ABA content without the expression of ABA biosynthesis genes have also been described in grape berries at softening and at veraison (Castellarin et al. 2016; Coelho et al. 2019). A cultivar-dependency was observed and it was proposed that the ABA increase at veraison might be caused by ABA import from other tissues or its lower turnover, which is supported by a decreased expression of ABA 8′-hydroxylases (Coelho et al. 2019). In BS-berries, the ABA content was slightly lower until veraison and higher thereafter (no significant values). This trend was in accordance with a strong increase in expression of *VviNCED2* in BS berries starting from EL35 till EL37 (Fig. 2d) as well as with the peak in expression of *VviNCED3* after veraison (Fig. 2e). RNAseq data confirmed the up-regulation of genes related to ABA biosynthesis [VIT_10s0003g03750 (*VviNCED2*); VIT_18s0001g10500 (*VviCYP707A4*); VIT_02s0087g00930 (*VviNCED4*, *VviCCD4a*); VIT_02s0087g00910 (*VviNCED4*, *VviCCD4b*)], ABA signaling [VIT_08s0058g00470 (*VviPYL4*); VIT_02s0012g01270 (*VviPYR1*)] and ABA catabolism [VIT_17s0000g02680 (*VviBGLU44*); VIT_06s0004g01430 (*VviBGLU12*)] after veraison, in BS berries (Fig. 2g). These expression profiles are in contradiction with ABA levels, which were gradually decreasing after veraison. However, strong increase of the main ABA storage metabolite ABA-GE indicated stimulation of ABA glucosylation (Fig. 2c), especially in BS berries. ABA-GE is a reversible glucoconjugate formed by UDP-glucosyltransferases as e.g. *VviUGT73B4,* which expression was strongly increased at veraison in healthy as well as BS berries (Fig. 2f). Nevertheless, the expression profile of *VviUGT73B4* in BS berries cannot explain the enhanced levels of ABA-GE observed after veraison, stabilization of UGT73B4 protein might be responsible for this effect. In BS berries, ABA was quickly conjugated into ABA-GE and in parallel ABA-GE might be hydrolyzed by beta-d-glucopyranosyl abscisate beta-glucosidases [*VviBGLU44* (VIT_17s0000g02680), *VviBGLU12* (VIT_06s0004g01430)], as both genes were induced in BS berries. This might contribute to the high number of ABA responsive genes strongly expressed in BS berries during the ripening phase (Fig. 2g). The irreversible catabolism of ABA is mediated by 8′-hydroxylases (CYP707A1) to form phaseic acid (PA) and dihydrophaseic acid (DPA). In our study the contents of PA and DPA were relatively high in H and BS berries before veraison, decreased at veraison, being lowest during the ripening phase (Fig. 2b). The level of DPA at veraison was significantly lower in BS berries, which corresponded to a slightly reduced expression of ABA 8′-hydroxylase *CYP707A1* (VIT_18s0001g10500) at EL35.

ABA signaling pathway is activated by ABA binding to receptors RCAR/PYR1/PYR1-LIKE (PYL) proteins and clade A protein phosphatases of type 2C (PP2C), which function as co-receptors. The complex formation results in PP2C degradation, releasing the associated protein kinases (SnRK2s) from PP2C inhibition (Ma et al. 2009; Nishimura et al. 2010). Among the genes differentially expressed in our study, ABA signaling genes at veraison were either not affected in BS berries or slightly repressed, whereas all of them were induced during the ripening phase (Fig. 2g), supporting the idea that ABA triggered gene expression is induced in BS berries after veraison. To conclude, ABA and ABA-GE along with the ABA signaling cascade are altered in BS-berries. We postulate that the reduced expression of the switch genes in BS berries at veraison (Savoi et al. 2019) is unlikely to be linked to ABA metabolites as a sole factor, in spite of the fact that several switch genes, which are modulated by ABA (Pilati et al. 2017) or belong to the ABA signal cascade (Savoi et al. 2019) were reduced in BS berries at veraison (Fig. 2g). The transcription profiles at EL36 and EL37 in BS berries resembled changes described for over-ripening berries (Cramer et al. 2014) and the post-harvest withering process (Zenoni et al. 2016), as recently discussed (Savoi et al. 2019). ABA has a central role in drought stress responses via the activation of ABA-dependent signaling pathways. It is interesting that several transcription factors are likewise activated by drought stress and BS (Savoi et al. 2017). Berry shrinking may be associated with stress responses via ABA function due to turgor loss. The elucidation of the differences in mechanism of drought stress responses, BS and post-harvest berry withering seems to be a prerequisite for understanding of BS symptoms during the berry ripening phase. The trigger for the induction of ABA biosynthesis genes at the transcriptional level as well as the physiological necessity to store high amounts of ABA-GE in vacuoles of BS berries remains unclear.

### Ethylene precursor ACC peaks in BS berries before veraison

**Fig. 3:**
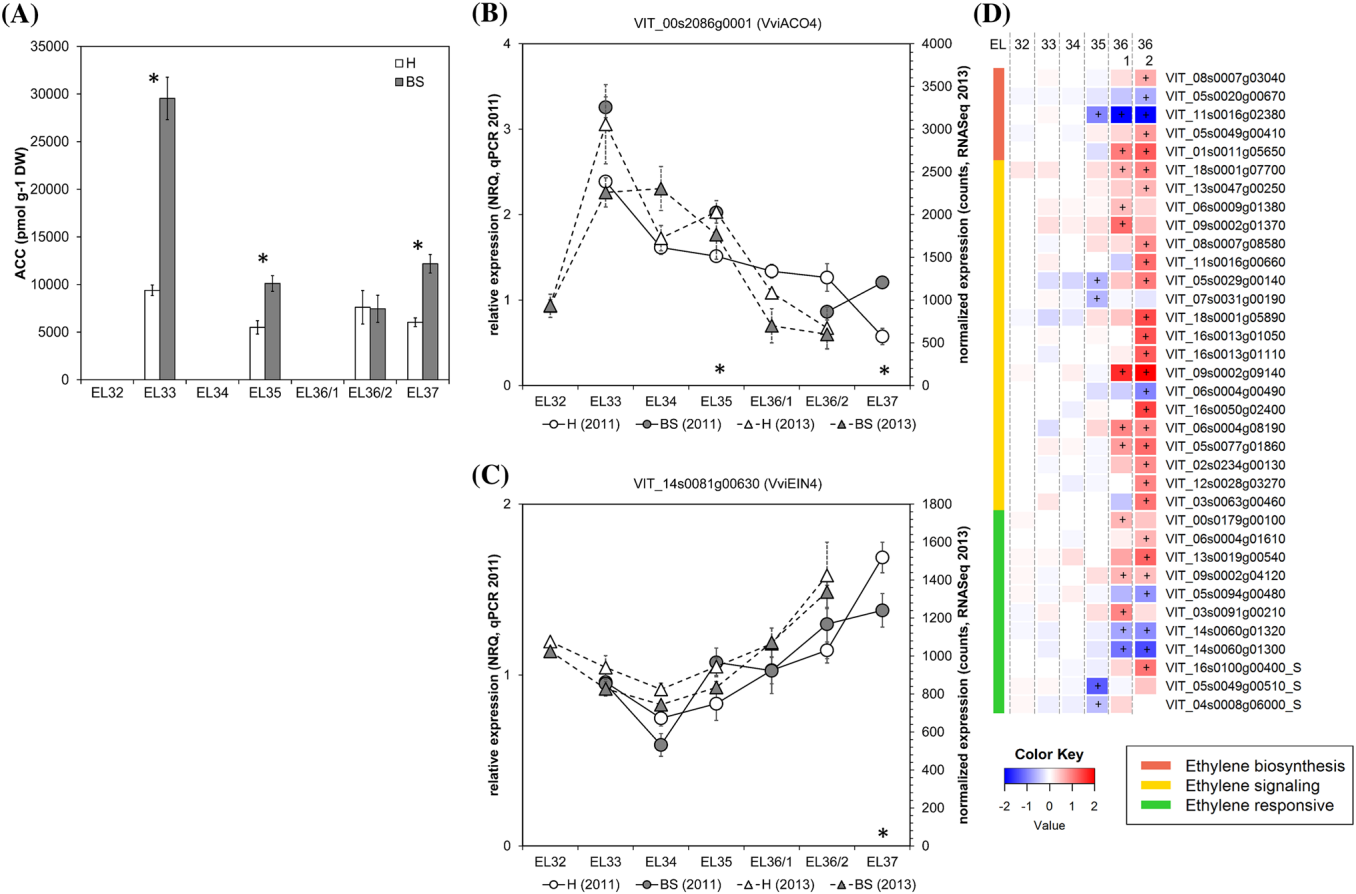
Results obtained for ACC content (**a**) and the expression of ethylene metabolism- and signal transduction-related genes in healthy (H) and berry shrivel (BS) grape clusters collected at six sampling dates (EL32, EL33, EL34, EL35, EL36/1, EL36/2, EL37) (**b**–**d**). **a** ACC (ethylene precursor) content in berry samples collected 2011. Expression of ethylene biosynthetic gene *VviACO4* (**b**) and signaling-related gene *VviEIN4* (receptor) (**c**) determined by qPCR (samples 2011) in comparison with RNAseq analyses (samples 2013). **d** RNAseq results on genes related to ethylene biosynthesis, metabolism, signaling, and responses in BS samples shown as logFC. All data are mean values ± standard error (*n* = 3 each). Veraison (V) is defined as the start of coloring (EL35) and first BS symptoms were determined at EL36/1. Statistical significant differences are indicated with an asterisk (2011) and a plus symbol (2013) (*P* < 0.05)

The ethylene precursor ACC was analyzed as a marker of ethylene production. ACC content in healthy berries did not differ among the four sampling time-points with no specific peak observed before or at the onset of ripening (Fig. 3a). Normally, in healthy berries ethylene peaks just before veraison 7–8 weeks after flowering with a duration of 1 (Muscat Hamburg) to 2 weeks (Cabernet Sauvignon) (Chervin et al. 2004; Sun et al. 2010). In our study, we possibly missed the ethylene peak in H berries as we did not analyze samples 1 week before veraison. Previous studies showed that the inhibition of ethylene accumulation had an effect on grape berry ripening and resulted in smaller berries, lower anthocyanin levels and higher acidity (Chervin et al. 2004), a symptomatology similar to BS. We determined an ACC peak in BS berries at EL33 about 14 days before veraison, which is 6 weeks after flowering and 1 week earlier as described in previous studies in healthy berries. The potential shift of the ACC peak in BS berries will be further studied to elucidate this cascade. ACC is a direct precursor of ethylene, its biosynthesis is the rate limiting step in ethylene formation. Additionally, ACC can be metabolized also to three conjugates (malonyl-ACC, γ-gametyl-ACC, jasmonyl-ACC), with yet unknown biological roles and ACC is transported in plants to enable distant ethylene responses. Recently, a debate started whether ACC can have signaling function per se in plants (Van de Poel and Van Der Straeten 2014). The ACC peak in BS berries might have led to an ethylene signal, but other options like conjugation and transport cannot be excluded and should be considered in future approaches. The RNASeq and qPCR data (Fig. 3b–d) showed non-uniform expression profiles in BS berries and cannot explain the observed ACC peak at EL33 in BS berries. Before veraison, no genes related to ethylene metabolism were significantly differently expressed (Fig. 3d) and *VviACO4* (Fig. 3b) was highly expressed at EL33 suggesting an active ethylene biosynthesis in both sample types. Several ethylene biosynthesis genes were suppressed (VIT_05s0020g00670, *VviSAMT1*; VIT_11s0016g02380, *VviACO1*) in BS berries after veraison, whereas other genes were induced (VIT_01s0011g0565, 1-aminocyclopropane-1-carboxylate oxidase). Similar situation was found in case of ethylene responsive genes, whereas the majority of the genes involved in ethylene signaling were higher expressed in BS berries (Fig. 3d) and e.g. *VviEIN3* (Fig. 3c). Recently, transcriptional biomarkers for the onset of ripening were identified in grapevine and individual ethylene response factors belonged to both negative as well as positive biomarkers (Fasoli et al. 2018), which may at least partially explain the observed undetermined expression profile in our study.

High ACC contents in BS berries led to the hypothesis that ethylene may trigger BS when applied before veraison. This hypothesis was tested by exogenous application of either ethephon (synthetic source of ethylene), ACC, AVG (aminoethoxyvinylglycine, inhibitor of ACC synthase, i.e. ethylene biosynthesis) or water and evaluation of BS symptoms of treated clusters. Treatment with ethephon led to a higher incidence of necrotic pedicels (similar to bunch stem necrosis) and berries dropped of the cluster very easily (Table 2). The application of ACC led to a slightly higher percentage of BS clusters (17% as compared to 6% in water controls), whereas the application of AVG did not change BS incidence. The observed phenotype of necrotic pedicels after ethephon treatment differed from BS symptoms. Clusters of this treatment reduced the fruit detachment force and promoted the development of dry stem scar, which was also observed after ethephon application in order to produce high quality stemless fresh-cut table grapes (Ferrara et al. 2016). Similar results were observed using ACC treatments and a combined ACC and methyl jasmonate one, which strongly promoted the production of ethylene by grape berries and was in combination more effective to stimulate abscission-related processes (Uzquiza et al. 2014). In contrast, the ACC treatment in our study produced the BS phenotype. The timing, the applied concentrations and interaction with other phytohormones [e.g. with auxin for fruit abscission (Kuhn et al. 2014a)] may affect the physiological responses during berry growth and ripening, having different impacts on ethylene content. These pilot data call for further tests to strengthen this conclusion and provide functional proof.

**Table 2:** Results of an exogenous application of chemicals affecting the ethylene metabolism in grape berries with the aim to induce berry shrivel

Treatment	Soluble solids (°Brix)	Grape clusters (each *N* = 30)		
		% Healthy	% BSN	% BS
Control	19.7 (± 0.75)	94	0	6
Ethephon	19.1 (± 0.91)	67*	33	0
ACC	19.0 (± 0.97)	80	3	17
AVG	19.8 (± 0.89)	94	0	6

Applied treatments were: control; exogenous application of water (10 mL, 10 days before veraison), Ethephon (1500 mg L^−1^; 2-chloroethylphosphonic acid, Bayer Crop Science), ACC [1000 mg L^−1^; 1-aminocyclopropane-1-carboxylic acid, VBC-30426 Testproduct (10%)] and AVG [250 mg L^−1^; l-α-(2-aminoethoxyvinyl)glycine hydrochloride, ReTain (15%), Valent BioScience]. Effects of the treatments were evaluated visually by BS incidence and additionally by determining soluble solids (°Brix) before harvest (13.09.2016). Soluble solids are shown as mean values ± standard deviation. Visual observation as percentage (%) BS, BSN and H clusters

*Berries drop off very easily

### Brassinosteroid biosynthesis was suppressed in ripening BS berries

**Fig. 4:**
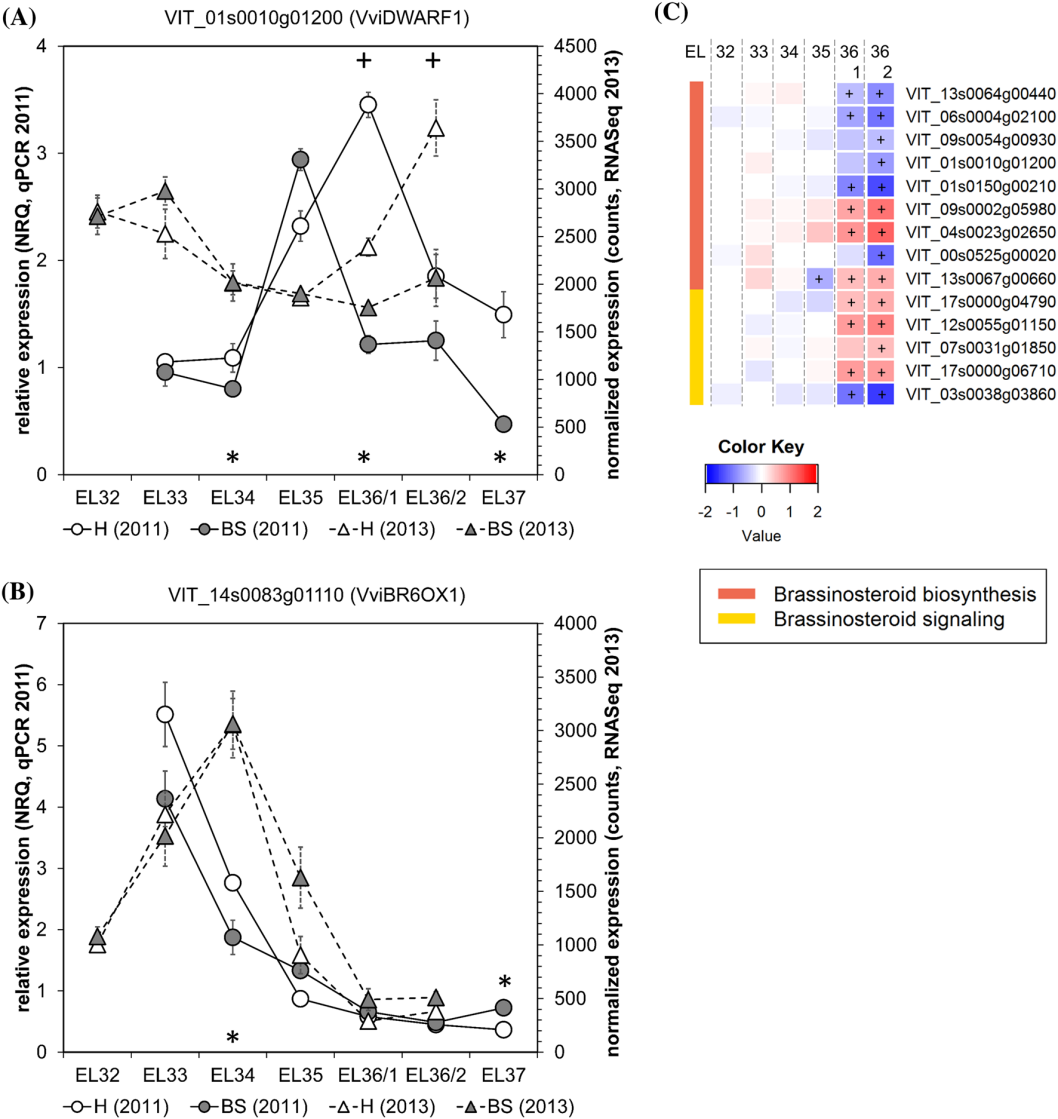
Brassinosteroid-related (BR) gene expression in healthy (H) and berry shrivel (BS) grape clusters collected at six sampling dates (EL32, EL33, EL34, EL35, EL36/1, EL36/2, EL37) (**a**–**c**). Expression of BR biosynthetic genes: **a***VviDWARF1* and **b***VviBR6OX1* determined by qPCR (samples 2011) in comparison with RNAseq analyses (samples 2013). **c** RNAseq results on genes related to BR biosynthesis and BR signaling in BS samples. All data are mean values ± standard error (*n* = 3 each). Veraison (V) is defined as the start of coloring (EL35) and first BS symptoms were determined at EL36/1. Statistical significant differences are indicated with an asterisk (2011) and a plus symbol (2013) (*P* < 0.05)

Brassinosteroids (BR) are a class of phytohormones with a diverse set of functions, e.g. promotion of cell expansion and cell elongation (together with auxin), stimulation of vascular differentiation, pollen tube formation and skotomorphogenesis as recently reviewed (Baghel et al. 2019). In grape berries BRs are increased at the onset of grape berry ripening and their exogenous applications can induce ripening (Symons et al. 2006). Therefore they are considered as early signals for ripening, possibly through ethylene content modulation (Ziliotto et al. 2012). Our gene expression analyses confirmed the induction of *VviBR6OX1* (brassinosteroid-6-oxidase) before veraison in both years in healthy grape berries (Fig. 4b). The values in BS berries were very similar, suggesting that BR biosynthesis is not affected before veraison which is in contrast to the situation observed after veraison. The expression of another BR biosynthesis gene *VviDWARF1* (22α-hydroxylase) was higher after veraison in healthy berries as compared with BS berries, which also exhibited a shift in expression peak towards veraison in 2011 (Fig. 4a). RNAseq data showed down-regulation of the expression of several BR biosynthesis genes in BS berries (Fig. 4d). The role of BRs during the grape berry ripening process was recently analyzed in more detail by applying exogenous BR (24-epibrassinolide) and the BR synthesis inhibitor brassinazole at the onset of veraison (Xu et al. 2015). Thereby exogenous BR application induced the activity as well as the expression of invertases and mono- and disaccharide transporters, while contrasting effects were obtained with brassinazole. Higher brassinolide contents led to a negative feedback to BR biosynthesis, resulting in down-regulation of the expression of *VviBR6OX1* and *VviDWARF1,* while the BR receptor *VviBRI1* was induced (Xu et al. 2015). BR biosynthesis seems to be reduced in BS berries, but currently we can only speculate about metabolite contents and their consequences for sugar accumulation. Previous studies reported an increased expression of invertases, hexose transporters, sucrose transporters and sucrose synthase in BS berries during the ripening phase as compared to controls, while tonoplast monosaccharide transporters, glycolysis and TCA cycle were suppressed (Savoi et al. 2019). Reduced BR biosynthesis could be the result of a negative BR feedback inducing enzymes and transporters for sugar accumulation. However without BR analyses the link remains speculation. Strikingly, BR biosynthesis is suppressed in BS berries, while many other phytohormone pathways are induced during the ripening phase.

### Inconsistent auxin responses

**Fig. 5:**
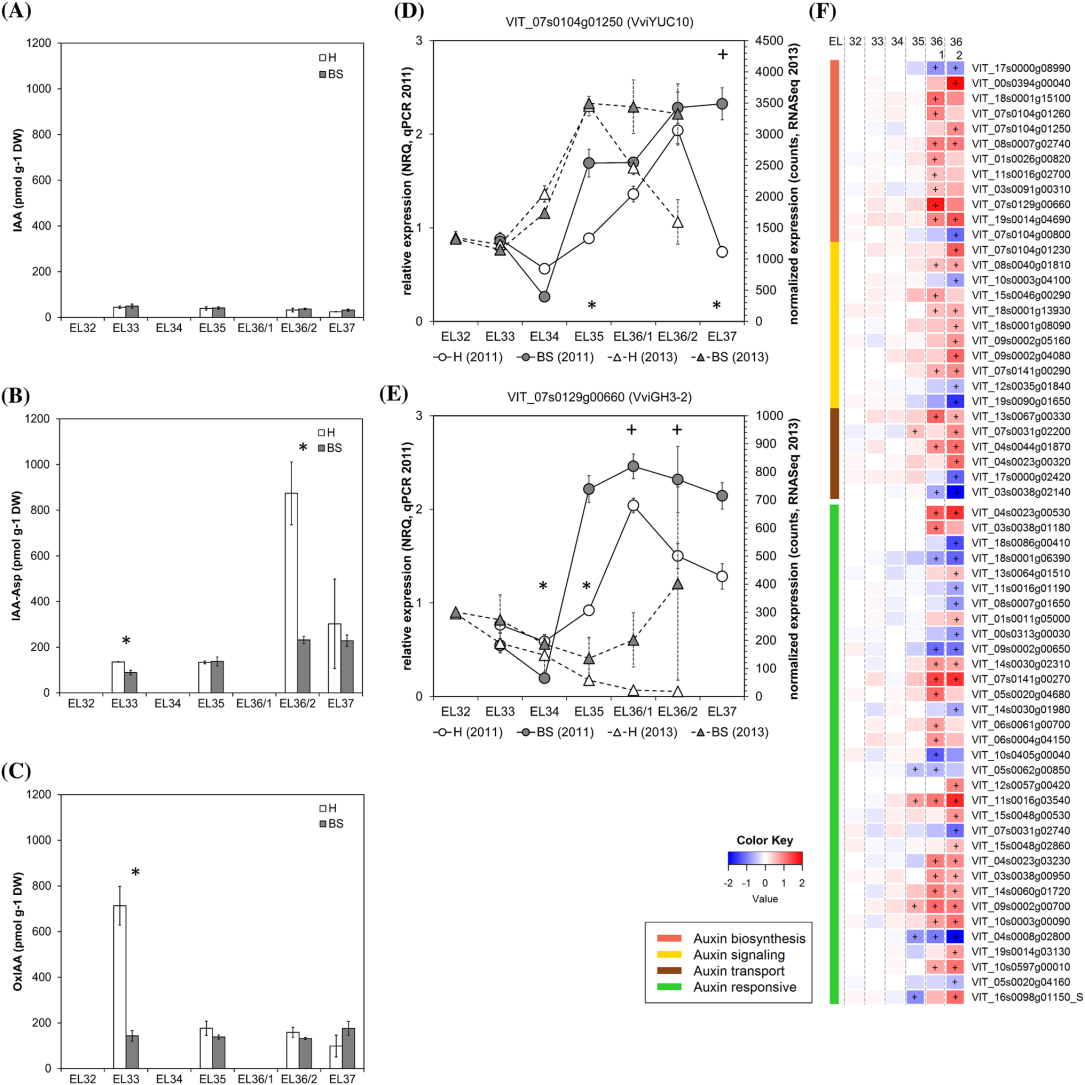
Results obtained from analyses of IAA and its metabolites (**a**–**c**) and expression of IAA metabolism- and signal transduction-related genes in healthy (H) and berry shrivel (BS) grape clusters collected at six sampling dates (EL32, EL33, EL34, EL35, EL36/1, EL36/2, EL37) (**d**–**f**). **a** Indole-3-acetic acid (IAA) content, **b** IAA-aspartate (IAA-Asp) content and **c** oxIAA content in berry samples collected 2011. Expression of IAA biosynthetic gene *VviYUC10* (**d**) and IAA metabolism-related gene *VviGH3-2* (**e**) determined by qPCR (samples 2011) in comparison with RNAseq analyses (samples 2013) (**g**). RNAseq results on genes related to IAA biosynthesis, signaling, transport, and responses in BS samples shown as logFC. All data are mean values ± standard error (*n* = 3 each). Veraison (V) is defined as the start of coloring (EL35) and first BS symptoms were determined at EL36/1. Statistical significant differences are indicated with an asterisk (2011) and a plus symbol (2013) (*P* < 0.05)

Auxins play an important role during the first growing phase of grape berries, but act as inhibitors of the ripening process in both climacteric and non-climacteric fruits. Low auxin levels are required at the onset of grape ripening, while conjugated forms of IAA usually increase with veraison (Bottcher et al. 2013b; Kumar et al. 2014). The application of NAA (1-napthalene acetic acid) to pre-veraison grapes delayed the onset of growth resumption, the accumulation of sugars and anthocyanins, the decrease in organic acid content and the increase in ABA levels (Bottcher et al. 2011, 2012). It seems that the entire berry ripening program is put on hold by auxin, which also delayed anthocyanin biosynthesis and switch gene expression in BS berries (Griesser et al. 2018; Savoi et al. 2019). Our data showed stable and not significantly different contents of IAA in H and BS samples (Fig. 5a). In healthy berries we observed a peak of 2-oxindole-3-acetic acid (oxIAA) at EL33 which is missing in BS berries (Fig. 5c). The complex interaction between auxin and ethylene was described (Muday et al. 2012), taking place also in pre-veraison grape berries (Bottcher et al. 2013b). Pre-ripening application of the ethylene-releasing compound Ethrel delayed the onset of ripening and enhanced the expression of genes for auxin biosynthesis and the amounts of IAA and IAA-Asp in grapes (Bottcher et al. 2013b). Other studies support the proposed cross talk between ethylene and auxin signaling in fruit ripening, although not all biochemical mechanism are known (Shin et al. 2016; Tadiello et al. 2016). Ethylene regulates root growth via effects on auxin biosynthesis, transport and signaling and ethylene as well as auxin regulate the transcription of key genes associated with the biosynthesis of both hormones (Fernie and Alseekh 2018; Muday et al. 2012). Whether the ACC peak in BS berries at EL33 actually influenced the auxin metabolism (e.g. oxIAA levels and induced auxin biosynthesis) needs to be determined as well as if auxin could influence ACC synthase at an early stage of berry growth. Experimental approaches are being conducted to decipher their interaction in grape berry ripening and maybe also in the sense of BS induction.

Auxin homeostasis in cells is tightly controlled and free IAA content is modulated by conjugation and/or oxidation (Ljung 2013). IAA can either be conjugated via ester linkages to glucose by UDP-glucosyl transferases or to amino acids by the Gretchen hagen3 (GH3) family of IAA-amido synthetases, or oxidized via IAA oxidase to form oxIAA, which seems to be the major catabolic pathway (Zhang and Peer 2017). Low auxin levels are necessary to start ripening as indicated by down-regulation of several auxin related and response genes at the onset of ripening (Bottcher et al. 2011; Fasoli et al. 2018). Only two genes, namely *VviIAA19* and *VviSAUR29,* were identified as positive biomarkers for the onset of ripening (Fasoli et al. 2018; Palumbo et al. 2014). Both genes, *VviSAUR29* (VIT_15s0048g02860) and *VviIAA19* (VIT_18s0001g08090), were only slightly induced at EL36/2 in BS berries and no difference in expression was observed at veraison. The expression analyses in BS berries provided rather a non-conclusive picture, as several auxin related genes reacted in opposite ways. The enhancing trend in the auxin biosynthesis could be observed in BS berries after veraison (Fig. 5f), while an increased levels of IAA-aspartate (IAA-Asp) were determined in H but not in BS berries after veraison, although an induction of *VviGH3-2* (VIT_07s0129g00660) (Fig. 5e), one of the genes responsible for the IAA conjugation, was detected. The expression pattern of *VviGH3-2* differed between the tested years (Fig. 5e). Recently a cultivar specificity of the expression profile of this gene was described (Coelho et al. 2019), which supports the pattern observed in 2013 in our study.

### Major decrease of the cytokinin iP in BS berries during the ripening phase

**Fig. 6:**
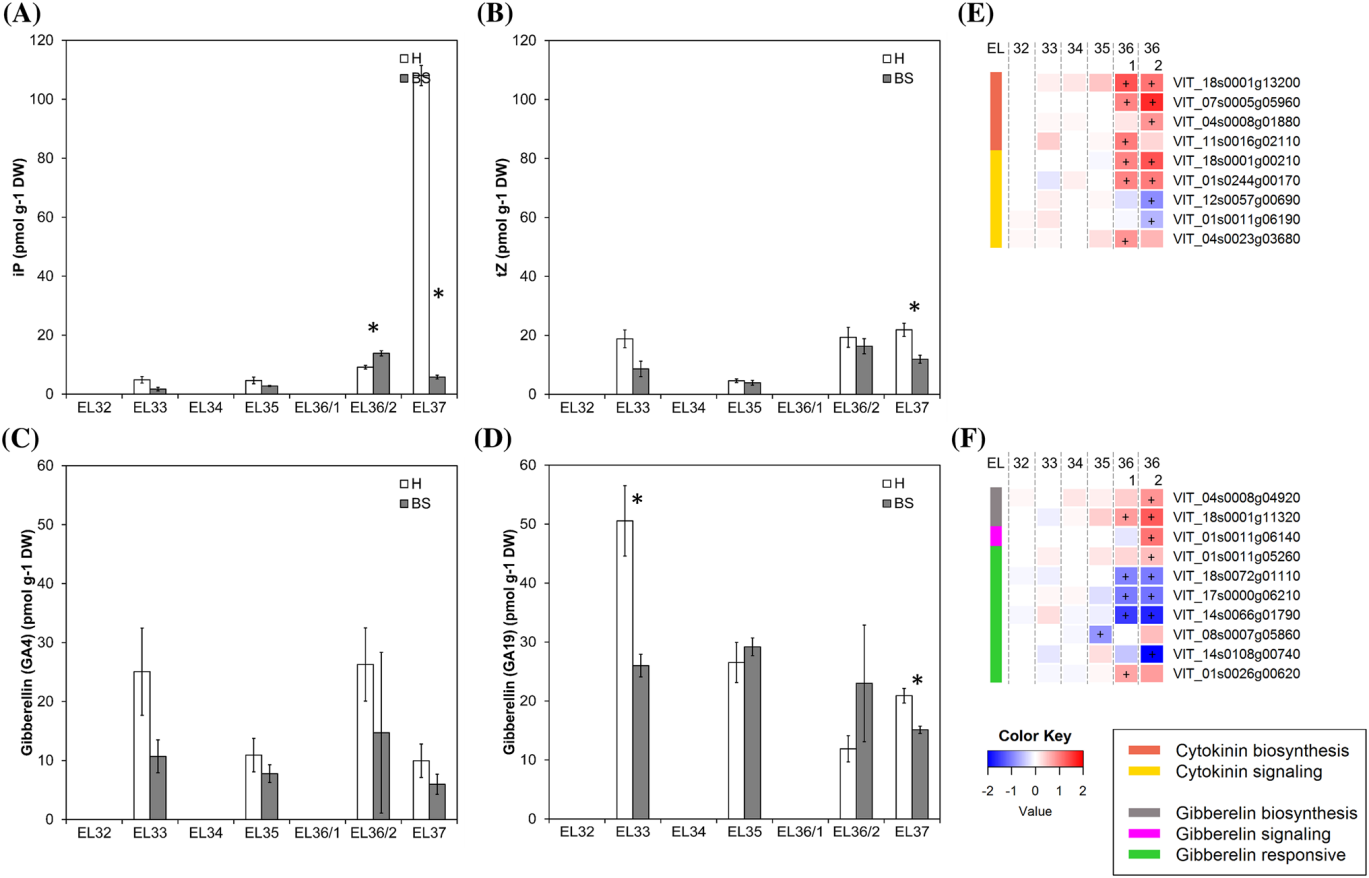
Results obtained from metabolite analyses of cytokinins and gibberellins (**a**–**d**) and expression of cytokinin and gibberellin metabolism- and signal transduction-related genes in healthy (H) and berry shrivel (BS) grape clusters collected at six sampling dates (EL32, EL33, EL34, EL35, EL36/1, EL36/2, EL37) (**e**–**f**). **a** Isopentenyladenine (iP) content, **b***trans*-zeatin (tZ) content, **c** GA_4_ (active gibberellin) content and **d** GA_19_ (gibberellin precursor) content in berry samples collected 2011. **e** RNAseq results on genes related to cytokinin biosynthesis and signaling and **f** RNAseq results on genes related gibberellin biosynthesis, signaling, and responses in BS samples shown as logFC. All data are mean values ± standard error (*n* = 3 each). Veraison (V) is defined as the start of coloring (EL35) and first BS symptoms were determined at EL36/1. Statistical significant differences are indicated with an asterisk (2011) and a plus symbol (2013) (*P* < 0.05)

The crucial cytokinin role is stimulation of cell division and differentiation, e.g. in shoot and flower development, female gametophyte development as well as vascular tissue and root meristem development and root nodule formation (Wybouw and De Rybel 2019). In plants, the active cytokinins are *trans*-zeatin (tZ), N6-(Δ^2^-isopentenyl)-adenine (iP), *cis*-zeatin (cZ), and dihydrozeatin (Haberer and Kieber 2002). Cytokinins peak after fertilization and during early fruit growth, stimulating cell division (Gillaspy et al. 1993). Their roles at later stages of fruit ripening are less described. Synchronous increase of iP together with soluble solids was observed in ripening berries supporting the important function of iP in the establishment and maintenance of sink strength (Bottcher et al. 2013a, 2015). In our study in total 22 cytokinin metabolites were analyzed (Table 1). We focused on iP and tZ as the most abundant active cytokinins in grapes. The level of tZ was transiently reduced around veraison (EL35) (Fig. 6b) which is in accordance with a previous report (Bottcher et al. 2015). The content of iP strongly increased in H berries at EL37 but not in BS berries (Fig. 6a). It has been proposed that the role of iP in ripening berries is related to the expansion driven growth after veraison and the high rate of sugar accumulation to establish and maintain sink strength, as shown for vegetative organs (Bottcher et al. 2015; Kuiper 1993). BS berries fail to accumulate sugars in high concentrations after veraison, although sugar accumulation starts, being stopped thereafter between 10 and 14°Brix (Griesser et al. 2012, 2018). Lower sink strength or loss of sink strength could be a possible explanation. The observed failed accumulation of iP in ripening BS berries could be one piece of information to support this conclusion. Nevertheless further studies are needed to investigate both the role of cytokinins, and especially of iP, in grape berries during the ripening phase in general and their role in BS symptom development related with sugar accumulation.

Gibberellins have a role in cell division and enlargement and higher contents of active gibberellins have so far been found in flowers and young developing fruits, whereas significant levels were not detected from veraison onwards (Bottcher et al. 2013a; Symons et al. 2006). GA_4_ and GA_19_ decreased during ripening and their lower contents were determined in BS berries, which correlated well with lower expression of gibberellin responsive genes in BS berries (Fig. 6c, d, f). No information on gibberellins during later grapevine ripening stages is available and the consequences for BS berries after veraison are unclear.

## Conclusion

The aim of this study is to characterize the processes that occur in BS phenotypes during the berry growth and ripening phases. Main symptoms of BS phenotypes are decreased sugar contents due to delayed sugar accumulation and weak sink strength while both rachis and pedicels remain green in color. In previous studies we showed that anthocyanin biosynthesis is delayed in ripening leading to lower anthocyanin contents (Griesser et al. 2018; Savoi et al. 2019) and the expression of 67 switch genes is reduced at veraison in BS berries (Savoi et al. 2019).

Here we show that many phytohormonal biosynthesis pathways (ABA, auxin, and cytokinin) are induced in BS berries after veraison on the transcriptional level, while ethylene and brassinosteroids are suppressed. One may question which process(es) keeps BS berries metabolically active during the ripening phase, as shedding or abscission of such berries would be resourceful. We describe two distinct phytohormone profiles in BS berry phenotypes: pre- and post-veraison. Firstly, an ACC peak about 2 weeks before veraison was determined in BS berries and the reciprocal ethylene-auxin crosstalk needs to be taken into consideration in a next step. The application of ACC pre-veraison led to BS symptoms while ethephon induced berry abscission. Temporal and spatial sensitivity towards phytohormone changes in grape berries throughout the ripening process and its consequences both in healthy and induced BS phenotypes are unclear. Secondly, we propose that the induction of several phytohormone pathways prevent fruit abscission as e.g. observed with bunch stem necrosis or sunburn, post-veraison. The similarities and differences in transcriptional patterns of ripening disorders and withering processes need to be determined as well as the role of iP (and possibly also of ABA-GE and IAA-Asp) in berry ripening as well as the consequences of its decreased accumulation for sink activity in berries. Sophisticated approaches and defined experiments are needed to decipher in detail the pivotal role of phytohormones in BS induction pre-veraison and in the development of BS symptoms after veraison. The presented study and previously published data provide valuable knowledge for these next steps.

## Electronic supplementary material

Below is the link to the electronic supplementary material.

Fig. S1 Samples were collected from a commercial vineyard in Lower Austria Climate (Antlasberg, Mailberg GPS coordinates 48.6667, 16.1833). Climatic conditions with monthly mean values for air temperature and rainfall were obtained from a neighbored ZAMG weather station. (DOCX 188 kb)

Table S1 List of candidate genes used for pPCR analyses and designed forward/reverse primers (DOCX 29 kb)

Table S2–S8 Selected phytohormone related genes differentially expressed in BS berries. Data basis for heatmaps in Fig. 2–6 (XLSX 247 kb)

## Data Availability

All raw transcriptomics reads have been deposited in NCBI Sequence Read Archive in the BioProject PRJNA436693 with SRA accession SRP134067 https://www.ncbi.nlm.nih.gov/bioproject/PRJNA436693/
