# Supplementary material for: Phytohormone profiles are strongly altered during induction and symptom development of the physiological ripening disorder berry shrivel in grapevine
Source: Plant Mol Biol. 2020 Feb 18;103(1):141–57. doi: 10.1007/s11103-020-00980-6 (PMC7170833; doi:10.1007/s11103-020-00980-6)
Supplement: Supplementary file 1 — Fig. S1 Samples were collected from a commercial vineyard in Lower Austria Climate (Antlasberg, Mailberg GPS coordinates 48.6667, 16.1833). Climatic conditions with monthly mean values for air temperature and rainfall were obtained from a neighbored ZAMG weather station. (DOCX 188 kb) [file 11103_2020_980_MOESM1_ESM.docx]

Figure S1


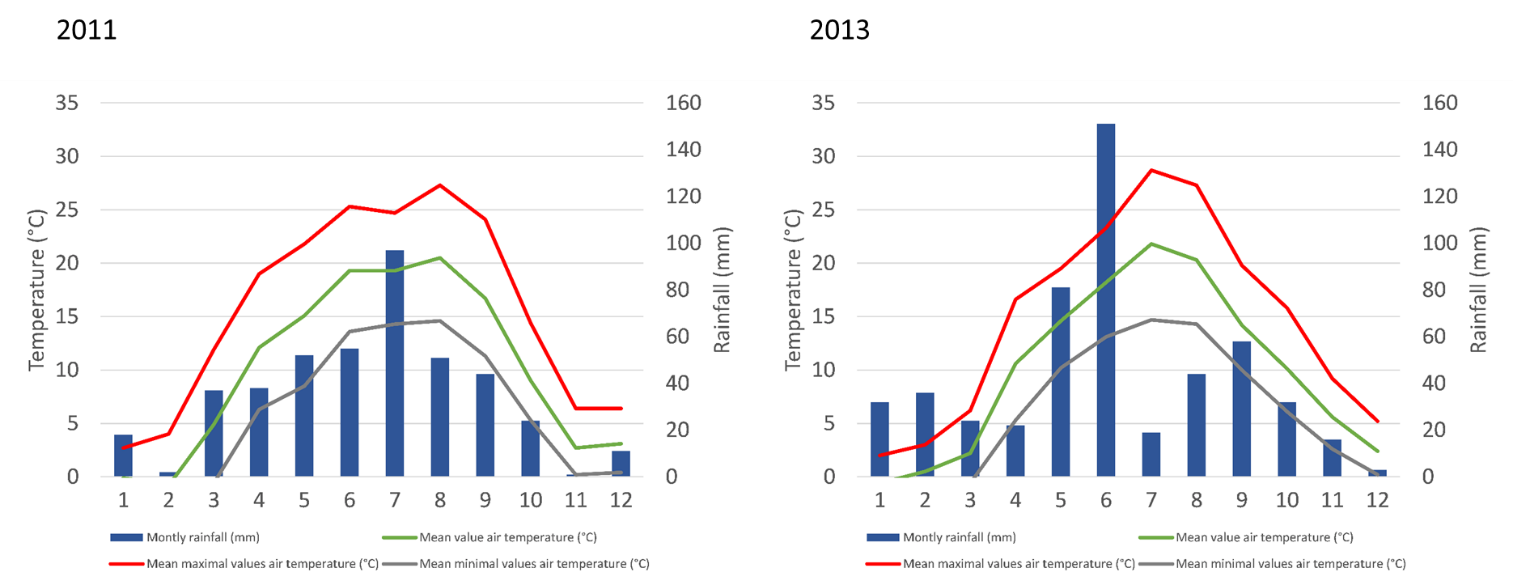


Figure S1: Samples were collected from a commercial vineyard in Lower Austria Climate (Antlasberg, Mailberg GPS coordinates 48.6667, 16.1833). Climatic conditions with monthly mean values for air temperature and rainfall were obtained from a neighbored ZAMG weather station.
