# Supplementary material for: Phytohormone profiles are strongly altered during induction and symptom development of the physiological ripening disorder berry shrivel in grapevine
Source: Plant Mol Biol. 2020 Feb 18;103(1):141–57. doi: 10.1007/s11103-020-00980-6 (PMC7170833; doi:10.1007/s11103-020-00980-6)
Supplement: Supplementary file 2 — Table S1 List of candidate genes used for pPCR analyses and designed forward/reverse primers (DOCX 29 kb) [file 11103_2020_980_MOESM2_ESM.docx]

Table S1

List of candidate genes used for pPCR analyses and designed forward/reverse primers

| **Accession number (12X V1)** | **Gene name** | **Forward primer (5´-3´)** | **Reverse primer (5´-3´)** |
| --- | --- | --- | --- |
| VIT_19s0093g00550 | VviNCED3 | CCTGGAGACGGTGGGTCGCT | AGCCACCGTTGAGGTATGGCTTCT |
| VIT_10s0003g03750 | VviNCED2 | GCAAGTGAACCTGGAAGCAGGCA | ATGGTTCCCCGCCGTACCTTTG |
| VIT_03s0063g00050 | VviUGT73B4 | TCGCCCCATGAGAAGGTGGGTT | TCGGGCGCTTGTTGGGTGTT |
| VIT_00s2086g00010 | VviACO4 | TGCAGTCATCTACCCAGCACCAGC | TTGGCCTGGAACTTCAAACCGGC |
| VIT_14s0081g00630 | VviEIN4 | GAAGTAGCAAAAGAATCCG | GCTTGCTGTCAGGGCTATG |
| VIT_07s0104g01250 | *VviYUC10* | CAGGAAACTGTCGCAATAGTGG | CAAGAACTATGTTGGGTATTGAGAGG |
| VIT_07s0129g00660 | *VviGH3-2* | ATCTACGAGCGCAAACAAGTCC | GTGTGAGTTGGTGCCAGTTGAG |
| VIT_14s0083g01110 | *VviBR6OX1* | TTGGGTTGTCCTACCACAGTC | TGGGGCTAATGAGAGCTAGAA |
| VIT_01s0010g01200 | *VviDWARF1* | CATGCACAGACAGCCCTAAA | CAACCACCCAAACAAAAACC |
| VIT_04s0044g00580 | *VviACT* | TGTGCTTAGTGGTGGGTCAA | ACCTGCTGGAAGGTGCTGAG |
| VIT_06s0004g03220 | *VviEF1* | gaactgggtgcttgataggc | ccggagtaaaagacacaacaaa |

Table 2: Quantification of phytohormones in healthy (H) and berry shrivel affected (BS) grape clusters. Samples were collected, 42, 55, 68 and 75 days after anthesis (DAA). Data presented are mean values on dry weight basis ± standard error (*n*=4 each) and statistical significant differences are indicated in bold letters (*P*<0.05).

|  |  | **DAA [mean value ± standard error; data in pmol g^-1^ DW]** | | | | | | | |
| --- | --- | --- | --- | --- | --- | --- | --- | --- | --- |
| **Metabolite** | **Abb.** | **42** | | **55** | | **68** | | **75** | |
|  |  | **H** | **BS** | **H** | **BS** | **H** | **BS** | **H** | **BS** |
| ***Auxins (AUX)*** |  |  |  |  |  |  |  |  |  |
| Indole-3-actic acid | IAA | 44.2 ± 9.6 | 49.1 ± 15.4 | 38.9 ± 12.6 | 41.1 ± 8.2 | 32.0 ± 14.6 | 36.4 ± 6.7 | 24.6 ± 1.6 | 31.4 ± 7.8 |
| IAA-aspartate | IAA-Asp | **135.4 ± 1.2 a** | **88.8 ± 17.4 b** | 133.6 ± 10.9 | 137.3 ± 33.9 | **873.7 ± 237.8 a** | **231.9 ± 27.1 b** | 302.2 ± 339.8 | 228.2 ± 42.3 |
| IAA-glutamate | IAA-Glu | 21.5 ± 10.4 | 9.3 ± 3.5 | 12.0 ± 6.3 | 10.7 ± 5.6 | 39.8 ± 27.6 | 19.2 ± 5.8 | 19.3 ± 15.0 | 18.7 ± 7.2 |
| oxo-IAA | OxIAA | **713.4 ± 146.6 a** | **143.6 ± 40.1 b** | 176.4 ± 53.8 | 138.0 ± 15.0 | 158.7 ± 37.7 | 131.8 ± 7.9 | 98.7 ± 82.5 | 175.8 ± 53.2 |
| Phenylacetic acid | PAA | 2184.4 ± 267.5 | 1554.4 ± 520.4 | 2600.1 ± 268.8 | 2310.5 ± 367.7 | 1296.0 ± 316.4 | 2004.7 ± 831.2 | 1079.2 ± 443.9 | 1201.6 ± 154.4 |
| IAA-glucose ester | IAA-GE | 5.7 ± 3.1 | 2.6 ± 2.8 | 2.3 ± 1.5 | 1.8 ± 0.8 | 0.8 ± 0.7 | 0.3 ± 0.1 | 0.8 ± 0.7 | 1.7 ± 0.4 |
| indolepyruvic acid | IPyA | 5.6 ± 3.8 | 5.2 ± 1.9 | 8.4 ± 7.0 | 4.2 ± 3.6 | 6.0 ± 1.2 | 6.6 ± 4.2 | 4.2 ± 3.4 | 9.3 ± 1.8 |
| Indole-3-acetamide | IAM | 3.7 ± 3.4 | 4.9 ± 2.8 | 4.3 ± 2.6 | 3.9 ± 3.8 | 4.9 ± 3.7 | 6.9 ± 3.7 | 1.4 ± 1.1 | 5.9 ± 4.7 |
| Indole-3-acetonitrile | IAN | 5.0 ± 2.5 | 2.2 ± 1.5 | 4.4 ± 2.7 | 0 ± 0 | 0 ± 0 | 0 ± 0 | 0 ± 0 | 0 ± 0 |
| ***Cytokinins (CK)*** |  |  |  |  |  |  |  |  |  |
| **active cytokinins** |  |  |  |  |  |  |  |  |  |
| Trans-zeatin | tZ | 18.8 ± 5.2 | 8.6 ± 4.5 | 4.6 ± 1.0 | 3.9 ± 1.5 | 19.4 ± 5.8 | 16.3 ± 4.4 | **21.9 ± 3.9 a** | **11.9 ± 2.3 b** |
| Trans-zeatin riboside | tZR | 22.2 ± 6.9 | 7.7 ± 1.2 | 6.3 ± 3.6 | 11.8 ± 1.1 | 12.2 ± 4.3 | 2.6 ± 0.7 | 1.9 ± 1.5 | 4.3 ± 3.3 |
| Cis-zeatin | cZ | 1.6 ± 2.1 | 1.1 ± 0.9 | 1.3 ± 0.6 | 1.1 ± 0.6 | 0.7 ± 0.6 | 1.1 ± 0.6 | **6.9 ± 2.8 a** | **0.4 ± 0.2 b** |
| Cis-zeatin riboside | cZR | 1.3 ± 0.4 | 1.0 ± 0.2 | 1.0 ± 0.5 | 1.1 ± 0.2 | 0.8 ± 1.0 | 0.8 ± 0.2 | **0.3 ± 0.1 b** | **1.3 ± 0.3 a** |
| Dihydrozeatin | dZ | 19.8 ± 6.8 | 8.4 ± 2.0 | 6.7 ± 3.6 | 3.4 ± 1.0 | **24.4 ± 9.0 a** | **3-4 ± 0.6 b** | 1.5 ± 0.9 | 4.4 ± 1.6 |
| Dihydrozeatin riboside | dZR | 15.6 ± 6.1 | 8.7 ± 2.7 | 10.9 ± 3.2 | 8.0 ± 1.0 | **20.6 ± 5.8 a** | **2.1 ± 0.8 b** | 1.4 ± 1.2 | 1.4 ± 1.2 |
| Isopentenyl adenin | iP | 4.9 ± 1.9 | 1.7 ± 1.0 | 4.6 ± 2.0 | 2.8 ± 0.4 | **9.1 ± 1.1 b** | **13.8 ± 1.5 a** | **108.0 ± 5.9 a** | **5.8 ± 1.1 b** |
| Isopentenyl adenosine | iPR | 1.4 ± 1.3 | 1.9 ± 0.1 | 0.7 ± 0.5 | 0.4 ± 0.6 | 0.1 ± 0.3 | 0.4 ± 0.4 | 0.1 ± 0.2 | 0 ± 0 |
| **deactivated cytokinins** |  |  |  |  |  |  |  |  |  |
| Trans-zeatin-7-glucoside | tZ7G | **11.2 ± 1.6** | **6.0 ± 0.9** | 3.6 ± 1.2 | 3.8 ± 1.7 | 3.5 ± 1.8 | 2.2 ± 0.7 | 1.8 ± 0.6 | 3.7 ± 2.1 |
| Trans-zeatin-9-glucoside | tZ9G | 2.0 ± 1.5 | 0.9 ± 0.5 | 1.1 ± 0.2 | 0.6 ± 0.3 | 0.8 ± 0.6 | 0.4 ± 0,.3 | 0.3 ± 0.4 | 0.2 ± 0.2 |
| Dihydrozeatin-9-glucoside | dZ9G | **11.3 ± 3.3** | **1.4 ± 0.5** | 3.7 ± 1.5 | 4.1 ± 1.8 | 7.6 ± 5.6 | 2.0 ± 0.3 | 1.8 ± 1.0 | 3.7 ± 2.8 |
| Isopentenyl-7-glucoside | iP7G | 6.3 ± 3.5 | 1.2 ± 0.4 | 2.9 ± 1.4 | 3.4 ± 1.7 | 1.6 ± 0.7 | 0.8 ± 0.5 | **0.6 ± 0.4 b** | **2.4 ± 0.7 a** |
| Isopentenyl-9-glucoside | iP9G | 0.1 ± 0.1 | 0.1 ± 0.0 | 0.1 ± 0.1 | 0.1 ± 0.0 | 0.2 ± 0.2 | 0.1 ± 0.1 | 0.0 ± 0.0 | 0.2 ± 0.3 |
| **storage cytokinins** |  |  |  |  |  |  |  |  |  |
| Trans-zeatin-O-glucoside | tZOG | **40.4 ± 6.6 a** | **15.7 ± 4.4 b** | 16.3 ± 7.6 | 21.9 ± 5.5 | 19.3 ± 9.2 | 8.7 ± 2.4 | **11.2 ± 3.7 b** | **26.0 ± 2.6 a** |
| Trans-zeatin riboside-O-glucoside | tZROG | 9.0 ± 5.9 | 2.5 ± 1.8 | 3.1 ± 1.7 | 3.7 ± 3.0 | 5.7 ± 1.5 | 5.1 ± 1.0 | 1.6 ± 1.8 | 4.5 ± 2.3 |
| dihydrozeatin riboside -O-glucoside | DRZOG | **18.9 ± 7.9 a** | **3.3 ± 2.1 b** | 11.2 ± 5.7 | 11.1 ± 3.8 | **43.3 ± 13.6 a** | **1.9 ± 2.1 b** | 1.3 ± 1.8 | 1.6 ± 1.8 |
| **cyotkinin precursors** |  |  |  |  |  |  |  |  |  |
| cis-zeatin-O-glucoside | cZOG | 1.2 ± 0.9 | 0.5 ± 0.3 | 0.3 ± 0.1 | 0.3 ± 0.2 | 0.4 ± 0.2 | 0.3 ± 0.3 | 0.3 ± 0.1 | 0.4 ± 0.4 |
| cis-zeatin riboside -O-glucoside | cZROG | 2.2 ± 2.9 | 1.5 ± 1.6 | 1.6 ± 1.3 | 2.0 ± 0.3 | 1.7 ± 1.2 | 2.4 ± 3.5 | 1.0 ± 0.8 | 1.5 ± 1.6 |
| Trans-zeatin riboside monophosphate | tZRMP | **13.1 ± 3.2 a** | **2.8 ± 1.1 b** | 4.2 ± 4.2 | 2.2 ± 1.5 | 2.8 ± 1.2 | 1.1 ± 0.8 | **1.9 ± 1.4 b** | **11.1 ± 3.5 a** |
| Cis-zeatin riboside monophosphate | cZRMP | 1.5 ± 1.6 | 0.1 ± 0.1 | 0.2 ± 0.2 | 0.5 ± 0.4 | 0.8 ± 0.5 | 0.4 ± 0.3 | 0.3 ± 0.2 | 0.4 ± 0.4 |
| Dihydrozeatin robiside monophosphate | DZRMP | 13.7 ± 3.2 | 18.5 ± 0.6 | 7.0 ± 2.4 | 4.1 ± 2.5 | **12.9 ± 7.3 a** | **0.5 ± 0.5 b** | 6.8 ± 1.7 | 2.6 ± 2.8 |
| Isopentenyl adenosine monophosphate | iPRMP | 10.4 ± 5.0 | 5.1 ± 3.5 | 2.3 ± 1.2 | 2.1 ± 0.8 | 2.1 ± 0.9 | 4.1 ± 3.8 | **4.2 ± 1.0 a** | **0.2 ± 0.1 b** |
| ***Salicylic acid (SA)*** |  |  |  |  |  |  |  |  |  |
| Salicylic acid | SA | 854.9 ± 114.3 | 950.2 ± 143.7 | **624.8 ± 25.5 b** | **683.0 ± 13.4 a** | 423.0 ± 123.5 | 658.0 ± 160.8 | **258.0 ± 8.8 b** | **470.3 ± 28.1 a** |
| ***Jasmonic acid (JA)*** |  |  |  |  |  |  |  |  |  |
| Jasmonic acid | JA | 168.9 ± 62.2 | 154.1 ± 28.0 | 74.1 ± 15.7 | 105.5 ± 14.8 | 32.3 ± 12.3 | 30.5 ± 0.7 | 15.5 ± 3.6 | 31.3 ± 10.4 |
| JA-isoleucine | Ja-Ile | 15.0 ± 5.2 | 23.2 ± 8.7 | 27.8 ± 8.0 | 27.3 ± 9.2 | 7.3 ± 2.7 | 9.9 ± 3.4 | 3.6 ± 2.4 | 8.7 ± 5.5 |
| cisOPDA | cisOPDA | 143.6 ± 64.3 | 62.6 ± 9.6 | **63.9 ± 11.0 b** | **162.5 ± 24.3 a** | 25.4 ± 10.7 | 21.7 ± 3.8 | 15.2 ± 17.8 | 17.3 ± 8.6 |
| ***Ethylene (ET)*** |  |  |  |  |  |  |  |  |  |
| 1-aminocyclopropane-1-carboxylic acid | ACC | **9386.8 ± 968.5 b** | **29534.6 ± 3864.4 a** | **5499.6 ± 1225.7 b** | **10108.4 ± 1431.7 a** | 7609.6 ± 3044.5 | 7454.6 ± 2467.7 | **6037.2 ± 802.3 b** | **12179.6 ± 1693.2 a** |
| ***Absissic Acid (ABA)*** |  |  |  |  |  |  |  |  |  |
| Abscisic acid | ABA | 5232.2 ± 1615.2 | 3411.0 ± 367.7 | 23697.9 ± 5038.7 | 16669.7 ± 4030.3 | 15125.0 ± 3114.5 | 20514.6 ± 2429.8 | 7084.5 ± 1247.1 | 10215.1 ± 1845.6 |
| ABA-glucose ester | ABA-GE | 4552.2 ± 264.6 | 4828.2 ± 252.0 | **12528.0 ± 473.9 a** | **6731.5 ± 621.8 b** | **11940.9 ± 1671.7 b** | **19460.0 ± 3475.0 a** | **9331.6 ± 1121.5 b** | **23818.3 ± 7126.2 a** |
| Phaseic acid | PA | 30.0 ± 8.1 | 44.1 ± 10.4 | 25.5 ± 4.4 | 26.2 ± 1.7 | 8.7 ± 1.8 | 9.7 ± 11.5 | 10.2 ± 1.7 | 4.0 ± 4.3 |
| Dihdyrophaseic acid | DPA | 4730.2 ± 1789.6 | 7166.2 ± 1385.1 | **1106.3 ± 268.0 a** | **694.1 ± 83.0 b** | 160.1 ± 22.7 | 201.8 ± 76.7 | **147.2 ± 35.5 a** | **83.4 ± 11.3 b** |
| ***Giberellic Acid (GA)*** |  |  |  |  |  |  |  |  |  |
| Giberellin 4 | GA4 | 25.1 ± 12.8 | 10.7 ± 4.8 | 10.9 ± 4.9 | 7.8 ± 2.6 | 26.3 ± 10.8 | 14.7 ± 23.6 | 10.0 ± 4.9 | 6.0 ± 3.0 |
| Giberellin 19 | GA19 | **50.6 ± 10.3 a** | **26.0 ± 3.3 b** | 26.5 ± 5.9 | 29.2 ± 2.6 | 11.9 ± 3.9 | 23.0 ± 17.1 | **20.9 ± 2.1 a** | **15.1 ± 1.1 b** |
